# Applicability of emission-based attenuation map for rapid CBF, OEF, and CMRO_2_ measurements using gaseous ^15^O-labeled compounds

**DOI:** 10.1186/s40658-015-0115-2

**Published:** 2015-05-30

**Authors:** Yukito Maeda, Nobuyuki Kudomi, Yasuhiro Sasakawa, Toshihide Monden, Koji Kato, Yuka Yamamoto, Nobuyuki Kawai, Yoshihiro Nishiyama

**Affiliations:** Division of Social and Environmental Medicine, Graduate School of Medicine, Kagawa University, 1750-1 Ikenobe, Miki-cho, Kita-gun, Kagawa, 761-0793 Japan; Department of Clinical Radiology, Kagawa University Hospital, Kagawa, 761-0793 Japan; Department of Medical Physics, Faculty of Medicine, Kagawa University, Kagawa, 761-0793 Japan; Department of Radiology, Faculty of Medicine, Kagawa University, Kagawa, 761-0793 Japan; Department of Neurological Surgery, Faculty of Medicine, Kagawa University, Kagawa, 761-0793 Japan

**Keywords:** Shortening PET examination, CBF, OEF, CMRO_2_

## Abstract

**Background:**

Cerebral blood flow (CBF), oxygen extraction fraction (OEF), and cerebral metabolic rate of oxygen (CMRO_2_) images have facilitated understanding of the pathophysiological basis of cerebrovascular disorders. Such parametric images can be rapidly, measured within around 15 min, using positron emission tomography (PET) with sequentially administered ^15^O-labeled oxygen and water. For further shortening, one option is to eliminate the transmission scan by applying an emission-based attenuation correction.

**Methods:**

The validity of the present method was tested by comparing parametric values with emission-based attenuation correction to those with transmission-based correction. This was applied to 27 subjects who were diagnosed with or without cerebrovascular disorders. All subjects received the rapid CBF/OEF/CMRO_2_ PET measurements. An emission-based attenuation map was generated by estimating the edge of the brain tissue contour on an obtained sinogram and by assuming the uniform tissue coefficient to be 0.1 cm^−1^. Then images were reconstructed, and parametric images were computed.

**Results:**

No difference was apparent between the emission- and transmission-based methods. Paired *t*-test showed no significant differences in CBF, OEF, or CMRO_2_ values between the emission- and transmission-based methods, except in the parietal and occipital and cerebellum and occipital regions, and the differences were less than 10%. The regression analysis showed a close correlation of *r* = 0.89 to 0.99.

**Conclusions:**

The present study revealed that the attenuation correction can be performed by the emission-based estimation method and clinical PET duration can be shortened for the CBF, OEF, and CMRO_2_ gas study.

## Background

Cerebral blood flow (CBF), oxygen extraction fraction (OEF), and cerebral metabolic rate of oxygen (CMRO_2_) images have facilitated the understanding of the pathophysiological basis of cerebrovascular disorders. These images have been quantitatively measured using positron emission tomography (PET) with bolus administrations of gaseous ^15^O-labeled oxygen (^15^O_2_), carbon dioxide (C^15^O_2_) or water (H_2_^15^O), and carbon monoxide (C^15^O), in the conventional three-step method [1-3]. These parametric images have been measured with separate emission scans for three tracers of C^15^O for cerebral blood volume (CBV), C^15^O_2_ for CBF, and ^15^O_2_ for CMRO_2_, with additional waiting times set between the scans in order to avoid contamination from the previous tracer on the PET data, requiring a relatively long duration of around 1 h.

Recently, the duration for measuring CBF, OEF, and CMRO_2_ has been shortened by using dual-tracer autoradiography (DARG) and, further, by applying dual-tracer basis function methods (DBFM) [4,5]. Both of these methods are characterized by sequential administration of dual tracers of ^15^O_2_ and C^15^O_2_ typically with a 3-min interval during a single PET scan. The DBFM method can shorten the total examination period to approximately 15 min while maintaining the image quality and quantitative accuracy [5]. In order to shorten the examination period even more, alternatives are to shorten the transmission (or a CT-based) scan [6] which is applied for the attenuation correction or to eliminate the transmission scan. For attenuation correction without the use of the transmission scan data, an emission-based attenuation map becomes necessary.

In this study, we estimated the attenuation map from measured emission scan data, namely, sequentially administered gaseous ^15^O_2_ and CO_2_ scan data. The feasibility of this method was tested by comparing the regional values of CBF, OEF, and CMRO_2_ obtained against those with attenuation maps from transmission scans in subjects with or without cerebrovascular disorders.

## Methods

### Subjects

The subjects were retrospectively selected from a clinical database in our hospital. All of them received PET examination due to suspected cerebrovascular disorders between February 2010 and June 2013. According to Powers’ classification of chronic hemodynamics compromised by occlusive cerebrovascular disease [7], hemodynamic impairment can be categorized into two stages. Stage I is defined as an increase in CBV in the hemisphere distal to the occlusive lesion, with normal CBF, OEF, and CMRO_2_. Stage II is characterized by reduced CBF and increased OEF with normal CMRO_2_. On the basis of this classification, we separated the subjects into the following three groups: Group-0 consisted of subjects diagnosed as being without significant disorder from PET parametric images, namely, without any apparent differences in the arterial territories between hemispheres in CBV, CBF, OEF, or CMRO_2_ images (*n* = 10, 10 males, weight = 64.3 ± 7.4 kg, age = 63.5 ± 11.2 years). Group-1 consisted of patients diagnosed with chronic stenosis or occlusion with elevated CBV (*n* = 6, 5 males and 1 female, weight = 65.4 ± 13.1 kg, age = 63.1 ± 14.5 years). Group-2 consisted of patients diagnosed with chronic stenosis or occlusion with reduced CBF, elevated OEF (*n* = 10, 6 males and 4 females, weight = 54.9 ± 15.6 kg, age = 54.7 ± 25.1 years). The study was approved by Kagawa University Ethics Committee.

### PET measurement protocol

PET acquisition was carried out in 2D mode using a PET scanner (ECAT HR+, Siemens-CTI, Knoxville, TN, USA). After a 300-s transmission scan with Ge/Ga rod source (three sources were set and last for 1 year, with the strength at the beginning being around 180 MBq), a static scan was started at 3 min after the inhalation of 2,000 MBq of C^15^O for 3 min. After at least 10 min for radioactive decay, ^15^O_2_ (3,000 MBq, 60-s duration) and then C^15^O_2_ (3,000 MBq, 60-s duration) were administered in sequence, at a 10.0-min interval during the single PET scan, which consisted of 39 frames for a total of 780 s (6 × 10 s, 6 × 20 s, 3 × 30 s, 3 × 120 s, 12 × 5 s, and 9 × 10 s). During the dynamic scan, blood was manually sampled (approximately 1 ml) through a catheter inserted in the right radial artery at the start of each scan frame. The radioactivity concentration in the blood samples was measured using an ARC-400 well counter (Aloka, Tokyo, Japan).

### Data processing

For generating an attenuation correction map by the emission-based method, we applied an edge detection technique [8] which is implemented in the scanner software [9]. Briefly, an edge contour on a sinogram was detected by setting a threshold. The contour on the sinogram was smoothed by retaining lower order Fourier coefficients, and the smoothed contour was transformed to the edge contour on a reconstructed image. Then an attenuation map was generated using the algorithm implemented in the scanner software, assuming the tissue attenuation coefficient value to be uniform [9]. To apply the method, we first summed the measured sinogram in the following three phases: total scan duration phase, from 20th (640 s) to the end of the scan phase (second tracer phase), and from 35th (720 s) to the end of the scan phase (phase after second tracer inhalation). The edge of the brain tissue region on each summed sinogram was defined by setting a threshold value. The threshold value was set as 0.1 of the maximum value on the summed sinogram. The tissue attenuation coefficient value applied was 0.1 cm^−1^, which was preliminarily obtained for ten subjects randomly chosen from the present data set.

Dynamic sinogram data were corrected for dead time in each frame in addition to detector normalization. Tomographic images were reconstructed from the corrected sinogram data by the filtered back-projection method with a Hann filter with 9 mm FWHM. Attenuation correction was applied by the generated emission-based map, as well as the transmission-based map. The reconstructed images consisted of a 128 × 128 × 63 matrix size with a pixel size of 1.71 mm × 1.71 mm and 2.45 mm and with 39 frames.

Measured arterial blood time activity curves (TAC) were calibrated to the PET scanner and separated to oxygen and water content while correcting for delay and dispersion simultaneously [4,10-14]. The separated blood TACs were used as input functions.

Two sets of CBF, OEF, and CMRO_2_ images were generated according to the DBFM formula [5], one being from the dynamic reconstructed image from the emission-based attenuation map and the other from the transmission-based one.

### Data analysis

We placed regions of interest (ROI) on each of the bilateral parietal, frontal, temporal, and occipital cortical regions, white matter, and cerebellar cortical regions, namely, 36 ROIs in total, in each subject on the obtained CBF image. The ROI shape and size used comprised a circle 8.6 mm in diameter. Values of CBF, OEF, and CMRO_2_ in the same ROIs were compared between the emission- and transmission-based images using regression analysis and Bland-Altman plot. The obtained values were also compared by the paired *t*-test. *p* < 0.05 was considered statistically significant.

## Results

**Figure 1:**
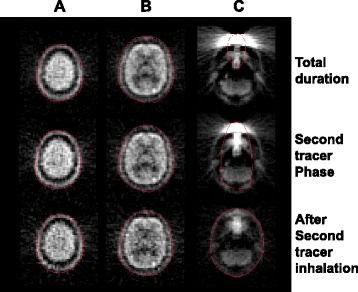
Automatically drawn edge (red line) on reconstructed image at the indicated levels. **(A)** centrum semiovale, **(B)** thalamus, and **(C)** cerebellum.

Figure 1 shows the detected and transformed edge contour on a reconstructed image estimated from the sinogram for three phases. At the level of (A) and (B), the drawn edges followed the face of the skull and were similar among the three summed phases. In the slice at the level (C) of the nasal cavity, the edges appropriately followed the contour of the head for the phase after the second tracer inhalation but did not follow it in the other phases.

The mean and SD of the CBF, OEF, and CMRO_2_ values derived by the transmission- and emission-based attenuation correction from the phase after second tracer inhalation are summarized in Tables 1, 2, and 3 for each subject group. Paired *t*-test showed significant differences in CBF, OEF, and CMRO_2_ values between the two methods, in the parietal, occipital, and cerebellar regions, with the differences in these regions amounting to around or less than 10%. For the emission-based method from summing the total duration and from the second tracer phase, both CBF and CMRO_2_ values were 5% to 10% higher than those for the transmission-based method in the frontal, temporal, and white matter and were lower in the parietal, occipital, and cerebellar regions but higher than those obtained by the emission-based method from the phase after second tracer inhalation.

**Table 1:** ** CBF, OEF, and CMRO**_2_ **values in Group-0 calculated applying the transmission- and emission-based attenuation correction**

		**Transmission**	**Emission**
CBF (ml/min/g)	Parietal	0.36 ± 0.08	0.34 ± 0.07*
	Frontal	0.40 ± 0.07	0.41 ± 0.07
	Temporal	0.43 ± 0.06	0.43 ± 0.07
	Occipital	0.44 ± 0.06	0.38 ± 0.06*
	Cerebellum	0.53 ± 0.09	0.51 ± 0.08*
	White matter	0.24 ± 0.04	0.24 ± 0.04
OEF	Parietal	0.47 ± 0.04	0.47 ± 0.05
	Frontal	0.46 ± 0.05	0.46 ± 0.05*
	Temporal	0.44 ± 0.05	0.44 ± 0.05
	Occipital	0.48 ± 0.04	0.47 ± 0.04*
	Cerebellum	0.50 ± 0.03	0.49 ± 0.03*
	White matter	0.43 ± 0.05	0.43 ± 0.05
CMRO_2_ (ml/min/g)	Parietal	0.027 ± 0.006	0.025 ± 0.004*
	Frontal	0.030 ± 0.004	0.030 ± 0.004
	Temporal	0.031 ± 0.005	0.031 ± 0.005
	Occipital	0.034 ± 0.006	0.029 ± 0.005*
	Cerebellum	0.044 ± 0.008	0.041 ± 0.007*
	White matter	0.016 ± 0.003	0.017 ± 0.003

Values are presented as means ± SD. *Significant difference (*p* < 0.05) between the transmission- and emission-based methods.

**Table 2:** **CBF, OEF, and CMRO**_2_ **values in Group-1 calculated applying the transmission- and emission-based attenuation correction**

		**Contra-lateral hemisphere**		**Ipsi hemisphere**	
		**Transmission**	**Emission**	**Transmission**	**Emission**
CBF (ml/min/g)	Parietal	0.43 ± 0.10	0.40 ± 0.07	0.42 ± 0.07	0.38 ± 0.05
	Frontal	0.44 ± 0.07	0.45 ± 0.08	0.39 ± 0.04	0.41 ± 0.05
	Temporal	0.45 ± 0.05	0.47 ± 0.04	0.43 ± 0.06	0.46 ± 0.05*
	Occipital	0.46 ± 0.04	0.42 ± 0.04*	0.44 ± 0.08	0.40 ± 0.07*
	Cerebellum	0.54 ± 0.08	0.55 ± 0.07	0.54 ± 0.09	0.56 ± 0.07
	White matter	0.22 ± 0.05	0.23 ± 0.04*	0.24 ± 0.04	0.25 ± 0.04
OEF	Parietal	0.46 ± 0.03	0.45 ± 0.03	0.44 ± 0.05	0.44 ± 0.05
	Frontal	0.44 ± 0.03	0.44 ± 0.03	0.47 ± 0.04	0.47 ± 0.04
	Temporal	0.42 ± 0.03	0.43 ± 0.04	0.44 ± 0.03	0.45 ± 0.03
	Occipital	0.47 ± 0.03	0.46 ± 0.03*	0.46 ± 0.04	0.45 ± 0.04*
	Cerebellum	0.48 ± 0.03	0.49 ± 0.04	0.48 ± 0.04	0.49 ± 0.04
	White matter	0.44 ± 0.04	0.44 ± 0.04	0.43 ± 0.06	0.43 ± 0.06
CMRO_2_ (ml/min/g)	Parietal	0.032 ± 0.006	0.029 ± 0.004*	0.030 ± 0.005	0.027 ± 0.005*
	Frontal	0.031 ± 0.007	0.032 ± 0.007	0.030 ± 0.005	0.031 ± 0.006
	Temporal	0.031 ± 0.003	0.032 ± 0.003	0.031 ± 0.003	0.033 ± 0.003
	Occipital	0.035 ± 0.002	0.030 ± 0.002*	0.033 ± 0.005	0.029 ± 0.004*
	Cerebellum	0.042 ± 0.003	0.043 ± 0.006	0.042 ± 0.005	0.043 ± 0.002
	White	0.016 ± 0.004	0.016 ± 0.003	0.016 ± 0.001	0.017 ± 0.001

Values are presented as means ± SD. *Significant difference (*p* < 0.05) between the transmission- and emission-based methods.

**Table 3:** **CBF, OEF, and CMRO**_2_ **values in Group-2 calculated applying the transmission- and emission-based attenuation correction**

		**Contra-lateral hemisphere**		**Ipsi hemisphere**	
		**Transmission**	**Emission**	**Transmission**	**Emission**
CBF (ml/min/g)	Parietal	0.45 ± 0.17	0.42 ± 0.14*	0.39 ± 0.16	0.36 ± 0.14*
	Frontal	0.44 ± 0.15	0.46 ± 0.17	0.36 ± 0.16	0.37 ± 0.16
	Temporal	0.48 ± 0.18	0.48 ± 0.18	0.40 ± 0.13	0.40 ± 0.13
	Occipital	0.47 ± 0.14	0.42 ± 0.11*	0.44 ± 0.13	0.39 ± 0.12*
	Cerebellum	0.51 ± 0.17	0.48 ± 0.17	0.56 ± 0.15	0.53 ± 0.16
	White matter	0.23 ± 0.07	0.24 ± 0.07	0.19 ± 0.05	0.20 ± 0.05
OEF	Parietal	0.48 ± 0.08	0.48 ± 0.09	0.52 ± 0.09	0.51 ± 0.09*
	Frontal	0.47 ± 0.07	0.48 ± 0.07	0.53 ± 0.13	0.53 ± 0.13
	Temporal	0.46 ± 0.08	0.46 ± 0.08	0.50 ± 0.10	0.50 ± 0.09
	Occipital	0.48 ± 0.07	0.47 ± 0.08	0.51 ± 0.08	0.50 ± 0.08*
	Cerebellum	0.50 ± 0.07	0.50 ± 0.06	0.50 ± 0.06	0.49 ± 0.05
	White matter	0.47 ± 0.10	0.48 ± 0.10*	0.54 ± 0.14	0.54 ± 0.13
CMRO_2_ (ml/min/g)	Parietal	0.035 ± 0.017	0.032 ± 0.014*	0.032 ± 0.017	0.029 ± 0.013*
	Frontal	0.033 ± 0.013	0.034 ± 0.015	0.029 ± 0.013	0.030 ± 0.012
	Temporal	0.035 ± 0.015	0.035 ± 0.014	0.032 ± 0.014	0.032 ± 0.012
	Occipital	0.035 ± 0.011	0.031 ± 0.008*	0.036 ± 0.014	0.030 ± 0.010*
	Cerebellum	0.040 ± 0.012	0.037 ± 0.011	0.044 ± 0.012	0.041 ± 0.011
	White matter	0.017 ± 0.005	0.018 ± 0.006*	0.016 ± 0.005	0.016 ± 0.005

Values are presented as means ± SD. *Significant difference (*p* < 0.05) between the transmission- and emission-based methods.

**Figure 2:**
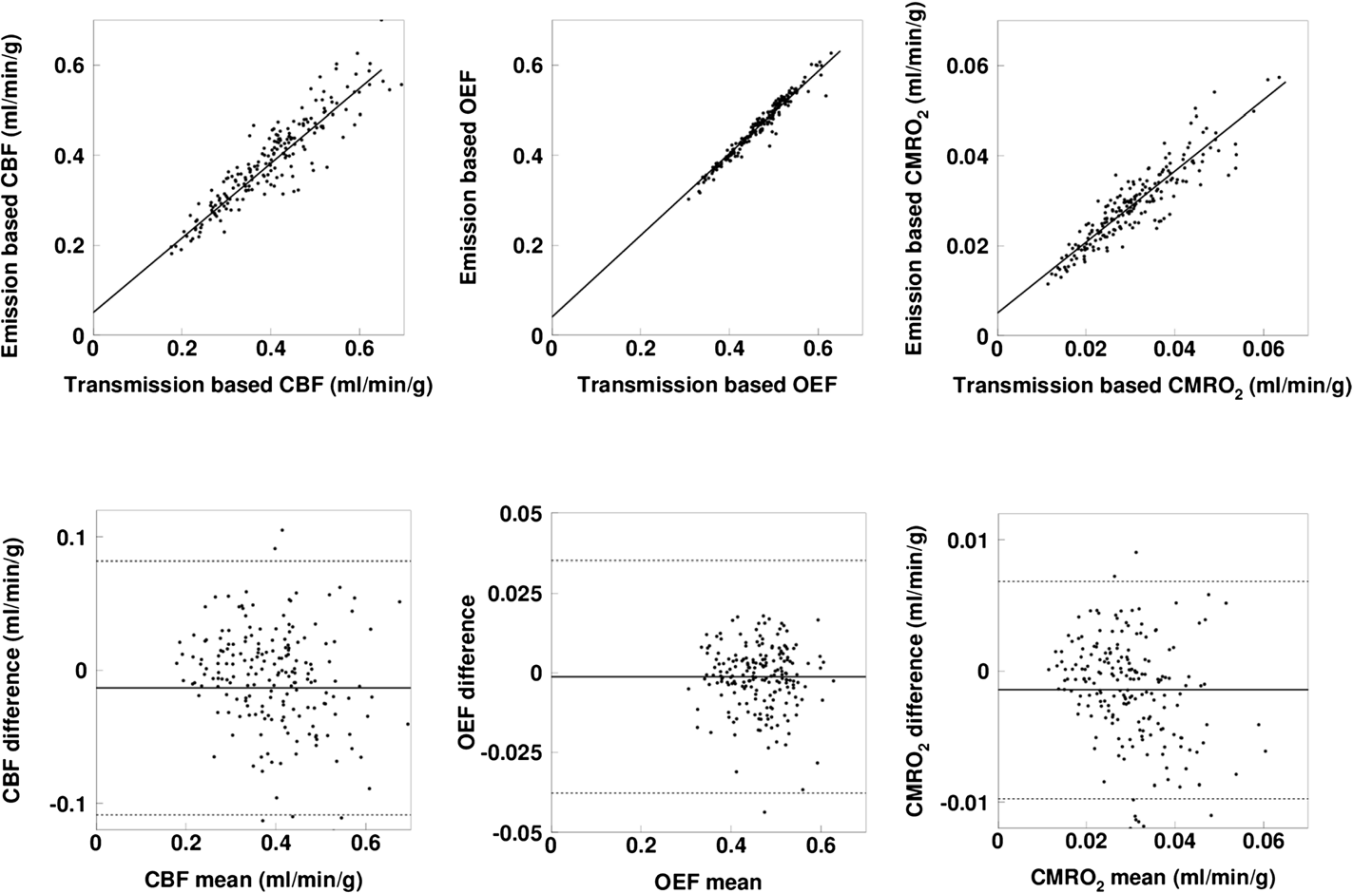
Relationship and Bland-Altman plot for Group-0 between the emission- and transmission-based methods. The regression lines are *y* = 0.83 *x* + 0.05 ml/min/g (*r* = 0.92), *y* = 0.91 *x* + 0.04 ml/min/g (*r* = 0.96), and *y* = 0.79 *x* + 0.005 ml/min/g (*r* = 0.92), for CBF, OEF, and CMRO_2_, respectively. In the Bland-Altman plot, solid and broken lines show mean of difference and its respective 2SD, respectively. Mean ± SD values are −0.013 ± 0.047 ml/min/g for CBF, 0.001 ± 0.018 for OEF, and 0.0014 ± 0.0042 ml/min/g for CMRO_2_, respectively.

**Figure 3:**
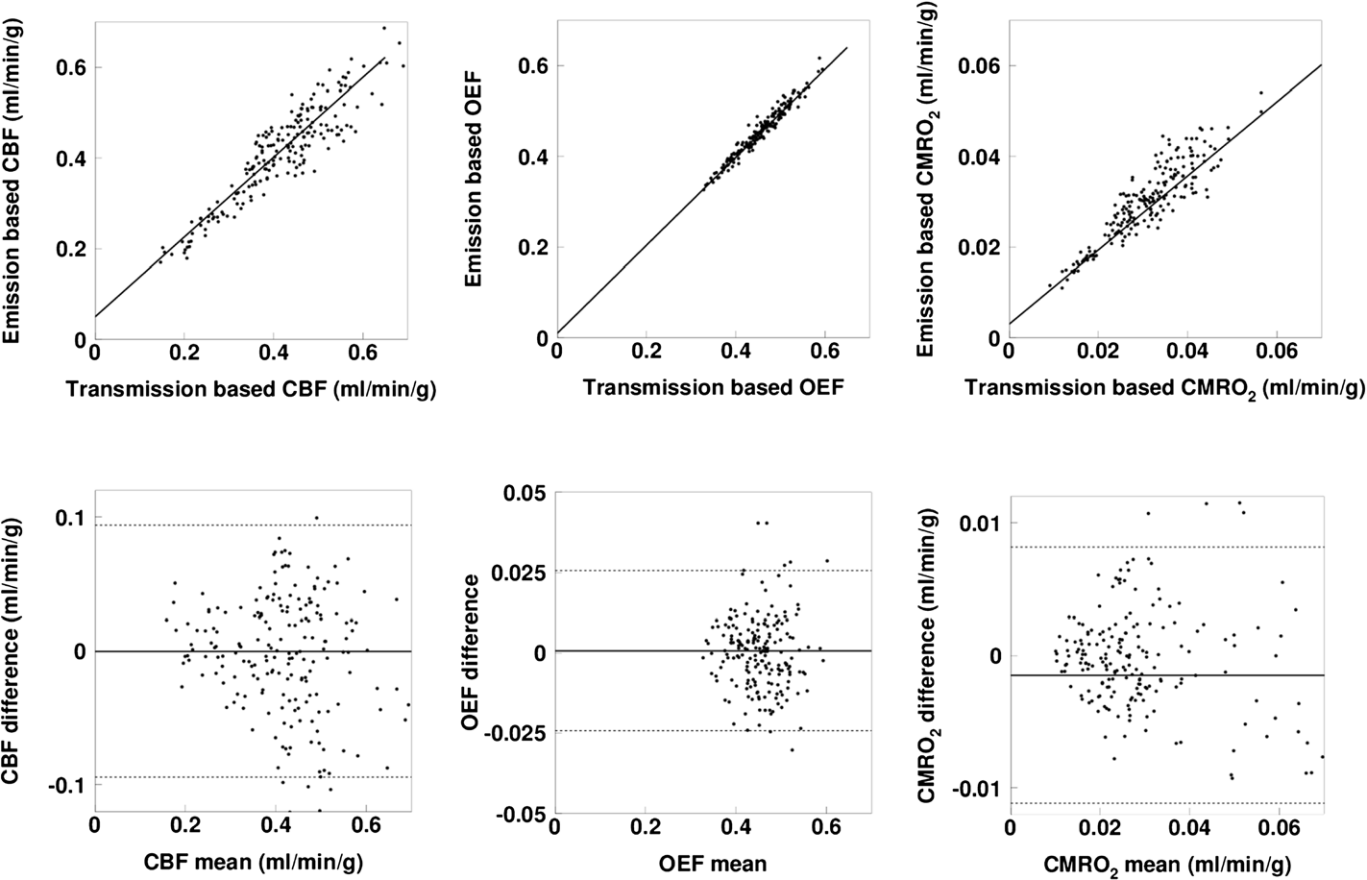
Relationship and Bland-Altman plot for Group-1 between the emission- and transmission-based methods. The regression lines are *y* = 0.88 *x* + 0.05 ml/min/g (*r* = 0.92), *y* = 0.97 *x* + 0.01 ml/min/g (*r* = 0.97), and *y* = 0.88 *x* + 0.003 ml/min/g (*r* = 0.89), for CBF, OEF, and CMRO_2_, respectively. In the Bland-Altman plot, solid and broken lines show mean of difference and its respective 2SD, respectively. Mean ± SD values are −0.000 ± 0.047 ml/min/g for CBF, 0.001 ± 0.012 for OEF, and −0.0005 ± 0.0043 ml/min/g for CMRO_2_, respectively.

**Figure 4:**
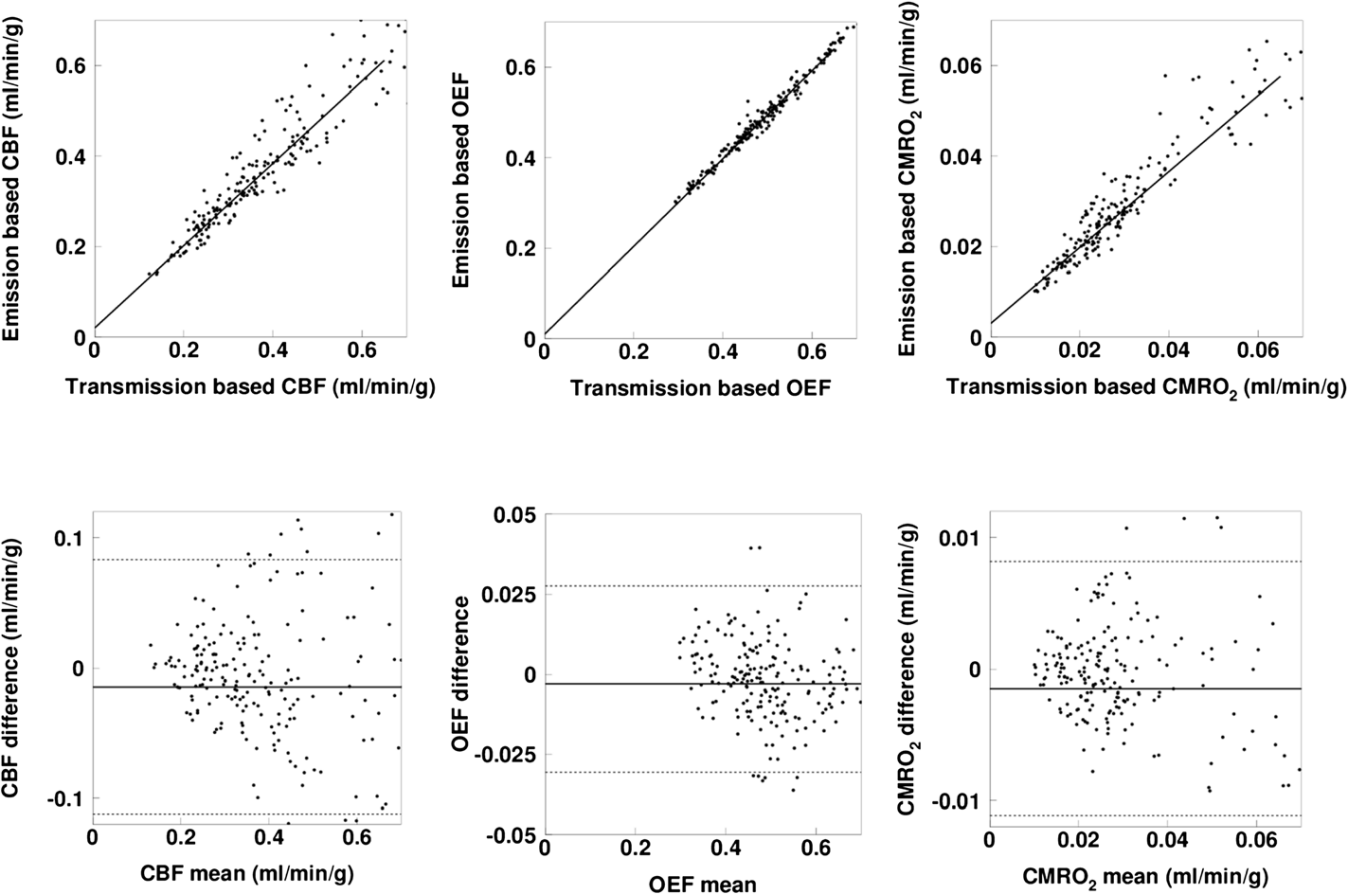
Relationship and Bland-Altman plot for Group-2 between the emission- and transmission-based methods. The regression lines are *y* = 0.91 *x* + 0.02 ml/min/g (*r* = 0.96), *y* = 0.97 *x* + 0.01 ml/min/g (*r* = 0.99), and *y* = 0.84 *x* + 0.003 ml/min/g (*r* = 0.90), for CBF, OEF, and CMRO_2_, respectively. In the Bland-Altman plot, solid and broken lines show mean of difference and its respective 2SD, respectively. Mean ± SD values are −0.014 ± 0.048 ml/min/g for CBF, −0.003 ± 0.013 for OEF, and −0.0015 ± 0.0048 ml/min/g for CMRO_2_, respectively.

The relationship and Bland-Altman plot in CBF, OEF, and CMRO_2_ between the emission- and transmission-based methods for Group-0, Group-1, and Group-2 are shown in Figures 2, 3, and 4. The regression analysis showed close correlations, with correlation coefficients of 0.89 to 0.99. The Bland-Altman plots demonstrated slight over- or underestimation, namely, −0.013 to −0.000 ml/min/g in CBF, −0.003 to 0.001 in OEF, and −0.0015 to 0.0014 ml/min/g in CMRO_2_, respectively, by the emission-based method.

**Figure 5:**
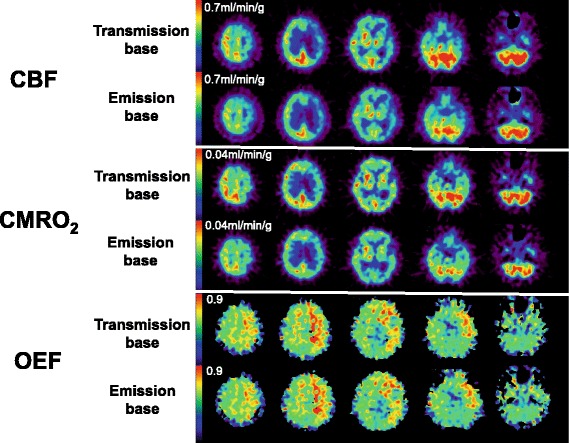
Representative view of CBF, OEF, and CMRO_2_ for Group-2 by transmission- and emission-based attenuation correction.

Representative sets of CBF, OEF, and CMRO_2_ images are shown in Figure 5. No apparent difference was seen between the emission- and transmission-based methods.

## Discussion

In the present study, we generated CBF, OEF, and CMRO_2_ images applying an emission-based attenuation map rather than transmission scan data. The differences between the emission- and transmission-based methods in the CBF, OEF, and CMRO_2_ values were around or smaller than 10%, though some of them differed significantly such as in the parietal, occipital, and cerebellum regions. The regression analysis showed close correlations with *r* = 0.89 to 0.99 between the methods, meaning that the regional contrast in the images was similar. These findings suggest that the present approach eliminating the transmission scan is applicable for clinical examination, particularly for patients with acute stroke.

The present application attainable here is as follows. First, several studies have already demonstrated emission-based attenuation correction like in the present method; however, they performed the scan by administering the tracer via only injection but not gas inhalation. It was not specified how the inhaled gas affected, particularly in the nasal cavity, the generated attenuation map by the emission-based method. The present study clarified the applicability for the gas inhalation method. Second, because of the considerable shortening of the total examination duration by eliminating the transmission scan, the patient’s burden, in particular, the need to stay still, would be reduced. Third, the applicability can be extended to the acute stroke patient, for whom the most rapid possible intervention is needed [15], and furthermore to assessments of hemodynamic change in a subject receiving a time-limited balloon occlusion test [16]. Bai et al. suggested that images without attenuation correction can give misleading, namely, a sphere in a thorax phantom was not visible without attenuation correction [17]. Careful attention is warranted to apply the present method when uniform structure cannot be assumed.

Some studies have focused on the elimination of transmission scans aside from PET/CT or PET/MRI. Weinzapfel and Hutchins in a CBF study during activation with and without transmission-based attenuation map found no significant difference between the methods [18]. Montandon and Zaidi demonstrated a method of template-based attenuation [19]. Kaneko et al. conducted a FDG study without transmission scan and found less adequate qualitative measurement in the uppermost and lowermost parts [20]. In generating the attenuation map by the emission-based method in the present study, we used the sinogram from 12 min to the end to define the edge of a brain contour and confirmed that the detected edge in the sinogram follows exactly the brain edge contour on a reconstructed image. When a sinogram from the total duration was used, the edge was blurred due to spillover of inhaled and exhaled ^15^O-labeled gas, resulting in an inappropriate attenuation map. Thus, extracting a phase not affected by the labeled gas is crucial for quantification of CBF, OEF, and CMRO_2_ in studies in which ^15^O-labeled gas compounds are administered.

The tissue coefficient value applied was 0.1 cm^−1^, which was obtained from the mean of measured attenuation maps for ten subjects randomly chosen from the present data set. For the ten values, mean and SD were 0.0996 ± 0.0013 cm^−1^, suggesting quite similar values across subjects. In fact, quite a similar value and variation of 0.099 ± 0.002 cm^−1^ was also demonstrated in a previous CBF study [18]. Some factors are potential sources of inaccuracy, such as sex, ethnic group, and age, and thickness of skull. When we applied the software for estimating attenuation map, the thickness of skull was not involved for the estimation, because that is not same across subjects and level of head. However, we do not anticipate significant errors attributable to these factors.

The CBF and CMRO_2_ values were obtained from uptake rate in water and oxygen phase and thus directly affected by the pixel value, thus degree of difference of these two parametric values between the methods were similar. The variations in OEF were less because that is computed as a rate of uptake rates in oxygen and water, meaning that the bias in the estimated pixel values was canceled.

There were significant differences in the CBF, OEF, and CMRO_2_ values between the emission- and transmission-based methods, such as in the parietal, frontal, cerebellum, and occipital regions. Such significant differences are likely attributable to some factors not taken into account in estimating the attenuation map, such as head-rest and skull for parietal region, and eyeball, muscle, ventricles, and bone, which could have different attenuation coefficients from that of the brain tissue, for occipital and cerebellar regions, and also any ^15^O-labeled gas retained in the nasal cavity and trachea. These factors appeared to bias the attenuation map in the emission-based method. Also, there is a methodological difference, namely, the transmission-based method is always affected by noise, while the other assumes uniform distribution of tissue coefficients, and thus is not affected by noise. This factor appeared as regional variation in attenuation maps from the transmission-based method. Such factors could result in regional dependency of over- or underestimation in the reconstructed pixel value and thus in estimated CBF, OEF, and CMRO_2_ values. In fact, the obtained CBF and CMRO_2_ values by the present method appeared lower than those by the transmission method in the parietal and occipital regions and cerebellum, due to the assumption that tissue coefficient values are uniform not only for brain but also for other tissues whereas they are in fact subject to bias from bone and soft tissue regions. In contrast, if the emission-based attenuation map was generated from a sinogram including a gas inhaling phase, the nasal cavity filled with ^15^O gas and its surrounding region might be considered brain tissue, resulting in overestimation of the pixel value and thus higher CBF and CMRO_2_ values. The CBF and CMRO_2_ values were obtained from the uptake rate in water and oxygen phase and thus directly affected by the pixel value. Thus, the degree of the difference in these two parametric values between the methods was similar. The variations in OEF were less marked because it is computed as a rate of uptake rates in oxygen and water, meaning that the bias in the estimated pixel values was canceled. The differences, however, were around or less than 10% in CBF and CMRO_2_ in the parietal, occipital, and cerebellum regions for the present emission-based method and were smaller than the SDs. As a whole, any apparent difference would not adversely affect any clinical determination of optimal treatments.

Recently, most PET systems are integrated PET/CT or PET/MRI scanners, and the 2D acquisition mode, which the present study applied, is not available, while only the 3D mode is. It would be important to refer the applicability of the present method to the 3D mode, but it would be important to directly test its validity. In theory, the present method might be extended to the 3D mode, because edge contour determination on the sinogram would be possible in the 3D mode like in the 2D mode. Then we could proceed with the same procedure. It should be noted that quantitative estimation of CBF, OEF, and CMRO_2_ has been achieved with 2D mode, while scatter coincidence events disturb the quantitative nature for 3D mode [20]. To overcome this, a hybrid dual-energy window method (HDW) [21,22] was applied and the validity of CBF, OEF, and CMRO_2_ images was demonstrated [23]. When scatter coincidence events interfere with the edge detection on sinograms, it would be possible to detect them on reconstructed images without attenuation correction, implementing the above HDW and proceeding with the following procedure. Thus, implementation of a hybrid scatter correction method would be essential for applying the 3D mode.

In the present study, the duration of transmission was 5 min, and the total true count was more than 50 M counts depending on the Ge/Ga rod source. In a previous study that estimated noise levels in CBF, OEF, and CMRO_2_ enhanced from transmission data, an *N*-index was introduced and the noise level was found not to be enhanced when the transmission true count was more than 40 M counts [6]. A simple comparison between the present and previous counts may not be feasible because of the different protocols as well as different PET scanners used, but, the count level in our study is higher, and thus, the transmission duration would be sufficient for the image quality. We also measured the *N*-index in our data set for those parametric images and found no significant deterioration in quality on parametric images.

We separated the present subjects into three groups with (two grades) and without cerebrovascular disorders and tested the validity of the present method, particularly in regions with elevated CBV and reduced CBF. The obtained parametric images showed that the CBF reduced and OEF elevated regions in the transmission-based method can also be identified with the emission-based method. The regional ROI values did not differ between the methods in any of the groups. These findings suggest that the present method with gas study is applicable to patients with cerebrovascular disorders, particularly acute stroke.

We set the time interval between the sequential administration of two tracers to 11 min, which is much longer than the 3 min the conventional methodology allows [4,5], in the present examination. The reason for this longer interval was due to limitations of the synthesizer system. The limitation is due to the need for a sequential supply of two radioactive compounds, namely, ^15^O_2_ and C^15^O_2_, within a short interval, including radio-synthesis, quality control, and purity examination before administration. An automated synthesis system able to operate under the same operation system as for the ^15^O-dedicated cyclotron would help to improve the logistics necessary in the procedures [24].

## Conclusions

The present study confirmed that the attenuation correction can be performed by the emission-based estimation method and clinical PET duration can be shortened for the CBF, OEF, and CMRO_2_ gas study.
